# The Flexible Peripheral Segments Modulate the Catalytic Activity of the FAD-Containing Monooxygenase EthA from *Mycobacterium tuberculosis*

**DOI:** 10.3390/biom16091333

**Published:** 2026-09-14

**Authors:** Dafeng Liu, Xieping Sun, Feng Yu, Hongying Song, Wenshuang Yao, Huashui Deng, Daoqi Song

**Affiliations:** 1 College of Biological Sciences and Technology, Yili Normal University, Yining 835000, China; 2 School of Life Sciences, Xiamen University, Xiamen 361102, China

**Keywords:** *Mycobacterium tuberculosis* (*Mtb*), FAD-containing monooxygenase EthA, mutation experiment, enzyme activity assay, molecular dynamics simulations

## Abstract

Tuberculosis (TB), caused by *Mycobacterium tuberculosis* (*Mtb*), remains a major global health concern, particularly due to the emergence of drug-resistant strains. FAD-containing monooxygenase EthA activates the antitubercular prodrug ethionamide (ETH) in *Mtb*. However, the structural and functional mechanisms of *Mtb* EthA are not fully understood. Here, we report an AlphaFold2-predicted structural model of *Mtb* EthA, validated using Ramachandran analysis (91.7% residues in favored regions) and a ProSA Z-score of −10.96. Based on the results of molecular docking, site-directed mutagenesis was conducted. We found that R207 is required for EthA activity. Alanine substitutions at T186, S208, and T210 resulted in 5.8- to 7.2-fold reductions in activity. Conversely, deletion of three peripheral segments (residues 137–176, 315–336, and 419–460) enhanced activity by 1.9-, 1.5-, and 1.2-fold, respectively. These results provide a structural framework for EthA function and suggest that flexible peripheral regions may constrain catalytic activity, offering insights into ETH activation and potential mechanisms of drug resistance.

## 1. Introduction

Tuberculosis (TB), caused by *Mycobacterium tuberculosis* (*Mtb*), remains a leading cause of mortality from a single infectious agent [1,2,3]. The emergence of drug-resistant strains, particularly multidrug-resistant (MDR) and extensively drug-resistant (XDR) TB, has significantly reduced the efficacy of first-line treatment regimens [4,5,6]. Ethionamide (ETH), a second-line thioamide, is widely used in combination therapies for MDR-TB [6,7,8,9]. ETH is a prodrug that requires intracellular activation to generate the active metabolite that inhibits InhA, the enoyl-acyl carrier protein reductase essential for mycolic acid biosynthesis [10,11,12,13]. This bioactivation is primarily mediated by EthA, a NADPH-dependent, FAD-containing monooxygenase of the Baeyer–Villiger monooxygenase family. EthA catalyzes the oxidation of ETH to an S-oxide intermediate, which subsequently reacts with NAD^+^ to form a covalent ETH-NAD adduct; this adduct represents the active inhibitory species targeting InhA.

**Figure 1:**
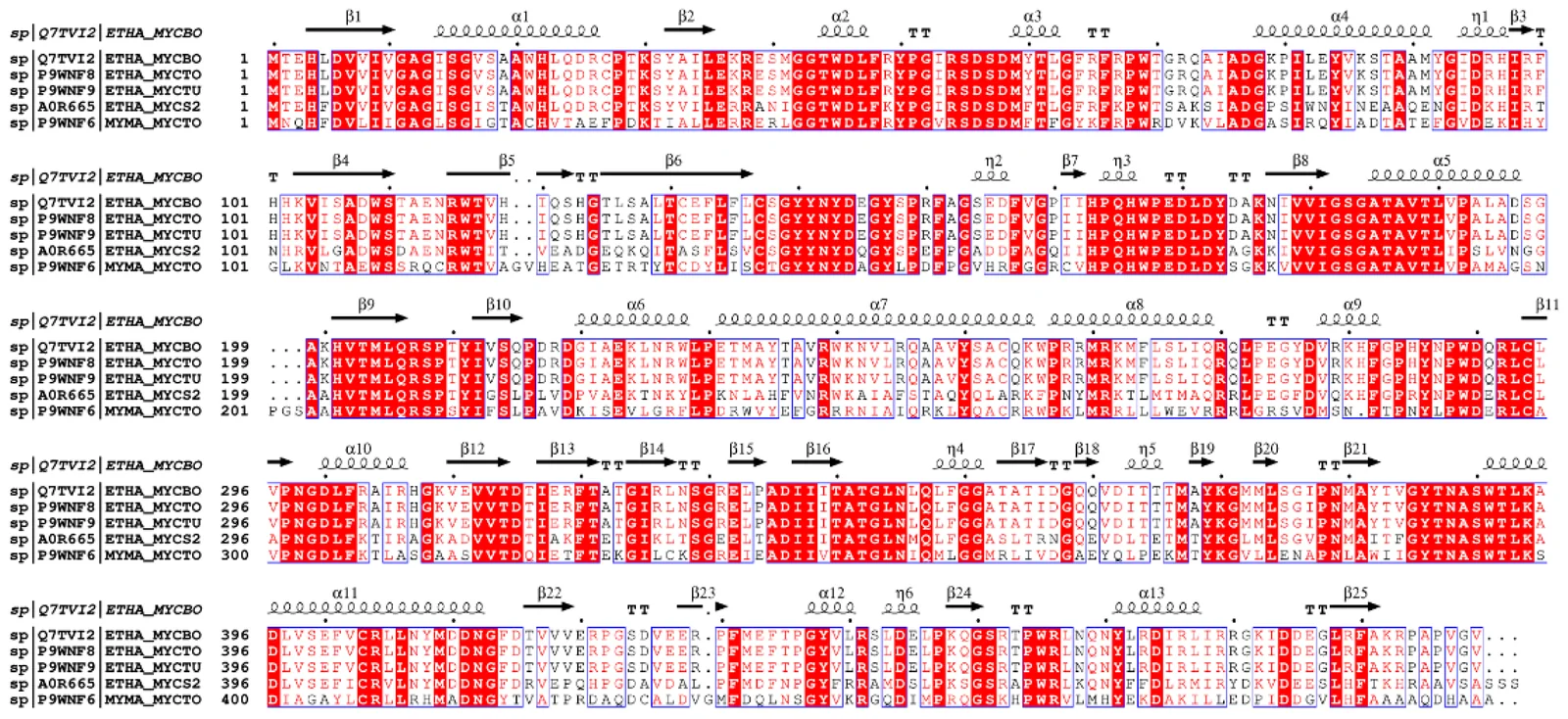
Sequence alignments of FAD-containing monooxygenases. The ClustalW default color scheme was applied, wherein conserved residues are assigned greater color intensity than non-conserved residues. The alignment includes the following reference proteins (UniProt accession and strain): Q7TVI2 (*Mycobacterium bovis* ATCC BAA-935/AF2122/97); P9WNF8 (*Mycobacterium tuberculosis* CDC 1551/Oshkosh); P9WNF9 (*Mycobacterium tuberculosis* ATCC 25618/H37Rv); A0R665 (*Mycolicibacterium smegmatis* ATCC 700084/mc(2)155); P9WNF6 (*Mycobacterium tuberculosis* CDC 1551/Oshkosh).

EthA expression is negatively regulated by EthR, a TetR/CamR-family transcriptional repressor that binds cooperatively to the EthA promoter region [11,12,14,15,16]. Loss-of-function mutations in EthR result in EthA overexpression and increased ethionamide (ETH) sensitivity, whereas EthR overexpression reduces EthA levels and confers drug resistance [6,17]. In clinical isolates, ETH resistance frequently arises from mutations in EthA, including missense, nonsense, and frameshift variants that abolish enzymatic activity [12,18]. Genomic analyses further indicate that most ETH-resistant strains harbor alterations in either InhA or EthA (including its 5′ untranslated region) (Figure 1), with high-level resistance often associated with multiple concurrent mutations [10,19,20]. Collectively, these findings underscore EthA as a key determinant of ETH susceptibility and a potential therapeutic target. However, the structural basis of EthA-mediated catalysis remains poorly characterized.

Herein, we used AlphaFold2 to predict a high-confidence structural model of Mtb EthA. The model was validated using Ramachandran plot statistics and ProSA Z-score analysis, confirming its geometric quality and overall fold reliability. Guided by the structural model, we performed site-directed mutagenesis to probe the functional roles of key active-site residues and peripheral regions. The results, detailed in Section 2.4 and Section 2.5, reveal both essential catalytic residues and modulatory peripheral segments that influence EthA activity. Molecular dynamics simulations showed that these regions exhibit elevated root-mean-square fluctuations, indicating increased backbone flexibility. These findings establish a structural framework for EthA-mediated ethionamide activation and suggest that modulation of peripheral loop flexibility represents a previously unrecognized mechanism regulating catalytic activity. This advances the molecular understanding of a key drug-activating enzyme in *Mtb* and may inform structure-based strategies to overcome ethionamide resistance.

## 2. Materials and Methods

### 2.1. Bioinformatics Analysis

Based on the amino acid sequence of EthA (FAD-containing monooxygenase; UniProt: P9WNF9) (Figures S1 and S2), multiple sequence alignments were performed using ClustalW with default parameters [21,22,23]. The alignment results were visualized using ESPript 3.0 [24].

### 2.2. Prediction and Assessment of the EthA Structure

The structural model of EthA was predicted using AlphaFold2 [25,26,27] based on the protein sequence (Figures S1 and S2) as input. Molecular visualization was performed using PyMOL v2.3.4 (https://www.pymol.org/2/) URL (accessed on 13 May 2026). Structural validation was carried out through geometric and statistical assessments. A Ramachandran plot generated via PDBsum [28,29,30,31,32] was used to evaluate backbone dihedral angles (φ/ψ) and assess stereochemical quality, identifying favored conformations and potential outliers, thereby providing quantitative support for model reliability. In addition, model quality was evaluated using ProSA (Protein Structure Analysis), a widely used validation tool that compares predicted structures against experimentally determined protein structures derived from X-ray crystallography and NMR spectroscopy [33].

### 2.3. Dynamic Light Scattering Characterization

To investigate the oligomeric state of EthA, dynamic light scattering (DLS) measurements were performed using a Dynapro DLS system (Malvern Zetasizer, Malvern, UK). Constructs, expression, and purification of EthA were carried out as previously described with minor modifications [11,12,14,18,19,34]. Purified EthA was concentrated to ∼2.6 mg/mL in buffer consisting of 50 mM Tris-HCl (pH 7.5), 150 mM NaCl, and 10% glycerol. The sample was centrifuged at 18,000 rpm for 5 min at 4 °C, and subsequently loaded into a 1 cm path length cuvette. Data were acquired over 30 runs with an equilibration time of 120 s. DLS data were analyzed using Zetasizer software (v6.20), generating regularized size distribution histograms. The hydrodynamic diameter (*D_h_*) was determined from the translational diffusion coefficient using the Stokes-Einstein equation: *D_h_* = *k_B_T*/(3*πηD_t_*), where *k_B_* is Boltzmann’s constant, *T* is the absolute temperature, *η* is the solvent viscosity, and *D_t_* is the translational diffusion coefficient [35]. The polydispersity index (PdI), which reflects the width of the size distribution, was also recorded, with values below 0.1 indicating a monodisperse sample. All measurements were performed in triplicate, and data are presented as mean ± SD. The hydrodynamic diameter was monitored throughout the analysis. While DLS provides valuable information about protein size and size distribution, it measures hydrodynamic radius rather than molecular mass directly; therefore, oligomeric state assignments based solely on DLS should be interpreted with caution.

### 2.4. Molecular Docking

Molecular docking of the cofactor NADPH (nicotinamide adenine dinucleotide phosphate; Figure S3) to the EthA binding site was performed using AutoDockTools 1.5.7 and AutoDock 4.2.6 [36,37,38,39,40]. The EthA structure was prepared by removing water molecules and adding polar hydrogen atoms and Gasteiger charges. The NADPH structure was prepared with energy minimization using the MMFF94 force field. Docking calculations were performed using the Lamarckian genetic algorithm (LGA) with the following parameters: a grid box of 60 × 60 × 60 points with a spacing of 0.375 Å centered on the predicted FAD-binding region; 100 independent docking runs; a population size of 150; a maximum number of 2.5 × 10^6^ energy evaluations; and a maximum of 27,000 generations. The final binding pose was selected based on the lowest binding energy cluster with the largest number of conformations (root mean square deviation < 2.0 Å). Following docking, results were analyzed and EthA-NADPH interactions were visualized using PyMOL v2.3.4 (https://www.pymol.org/2/). The calculated binding energy was −5.94 kcal/mol, which represents a computational estimate of the binding affinity [38,41,42]. It is important to note that this docking score is an in silico prediction and should be interpreted as a qualitative indicator of favorable binding rather than an absolute measurement of binding strength. The docking analysis was used primarily to guide the selection of residues for site-directed mutagenesis and to provide structural context for understanding the EthA-NADPH interaction, rather than to quantitatively assess binding affinity.

### 2.5. Enzymatic Activity Assay

Enzymatic activity of EthA and its mutants (Table S1) was determined using a modified version of a previously described protocol [11,12,14,18,19]. Activity was monitored by measuring the formation of ethionamide S-oxide (ETA-SO) at 350 nm over 5–10 min. The molar extinction coefficient of ETA-SO under the assay conditions was determined experimentally to be 6000 M^−1^ cm^−1^. All enzyme samples (wild-type and mutants) were normalized based on two complementary approaches: (1) protein concentration, determined by the Bradford assay using bovine serum albumin as a standard; and (2) FAD content, measured by absorbance at 450 nm (ε_340_ = 11,300 M^−1^ cm^−1^) to ensure consistent flavin incorporation. The final enzyme concentration in the assay was adjusted to 500 nM FAD for all samples. Activity values are reported as specific activity (nmol ETA-SO formed/min/nmol FAD) to account for potential differences in FAD occupancy between the wild-type and mutant proteins. This normalization strategy ensures that the observed activity differences reflect changes in catalytic properties rather than variations in protein amount or cofactor incorporation. To validate the assay, phenylacetone (250 μM), a known substrate of Baeyer–Villiger monooxygenases [43], was used as a positive control, with activity measured by monitoring NADPH consumption at 340 nm (ε_340_ = 6220 M^−1^ cm^−1^). The specific activity values obtained from the positive control experiments (8.1 nmol NADPH consumed/min/nmol FAD) were within the expected range based on published reports for related Baeyer–Villiger monooxygenases, confirming the reliability of our experimental conditions and enzyme preparations. The reaction mixture contained 50 mM Tris-HCl (pH 7.5), 100 mM KCl, catalase (100 U/mL), superoxide dismutase (100 U/mL), bovine serum albumin (0.1 mg/mL), and an NADPH-regenerating system comprising glucose-6-phosphate dehydrogenase (2 U/mL), glucose-6-phosphate (250 mM), and NADP^+^ or NADPH. The enzyme was typically used at a final concentration corresponding to 500 nM FAD. Ethionamide (Figure S4) stock solutions (25 mM) were freshly prepared in acetonitrile, with the final solvent concentration not exceeding 1% (*v*/*v*). All stock solutions were maintained on ice prior to use. Reactions were initiated by addition of ETA to the sample cuvette containing the enzyme and reaction mixture, while an equal volume of acetonitrile was added to the reference cuvette. The reference contained all components except enzyme and substrate, enabling correction for background absorbance, including NADPH. Prior to substrate addition, both sample and reference cuvettes were equilibrated at 37 °C for 2 min.

### 2.6. Molecular Dynamic (MD) Simulations

Molecular dynamics simulations were performed using GROMACS 2022.3 [44] with the AMBER99SB-ILDN force field [45] and the TIP3P water model, based on the predicted EthA structure. Long-range electrostatic interactions were treated using the particle mesh Ewald (PME) method [46,47], with a 1.2 nm cutoff applied to both electrostatic and van der Waals interactions. The Verlet cutoff scheme was used, and neighbor lists were updated every 10 steps. The system was maintained at 310 K and 1 bar using the Nose-Hoover thermostat [48] and Parrinello-Rahman barostat [49], with coupling constants of 0.4 ps and 0.1 ps, respectively, under periodic boundary conditions. Prior to production runs, energy minimization (steepest descent) was followed by NVT (100 ps) and NPT (100 ps) equilibration. Production simulations were performed for 200 ns with a 2 fs timestep. To assess convergence and reproducibility, three independent replicate trajectories were generated, each starting with different initial velocity assignments. The RMSF (root mean square fluctuation) profiles from the three replicates showed high consistency (Pearson correlation coefficient > 0.85), indicating that the flexibility patterns reported are reproducible. Trajectory analyses, including RMSD (root mean square deviation) and RMSF calculations, were carried out using GROMACS tools and visualized with xmgrace.

### 2.7. Antibacterial Activity Test

Mid-log phase cultures of *Mtb* H37Rv (ATCC 25618) were standardized to 1.73 × 10^7^ CFU/mL in 7H9 medium supplemented with 10% OADC (oleic acid-albumin-dextrose-catalase) and 0.05% Tween 80, and then diluted to a final inoculum of 10^6^ CFU/mL for assays. Two-fold serial dilutions of ETH were prepared in 96-well plates. Isoniazid (INH) and rifampicin (RIF) were used as positive control drugs to validate the assay methodology; two-fold serial dilutions of INH and RIF were prepared in parallel with the ETH dilutions. The minimum inhibitory concentration (MIC) was defined as the lowest ethionamide concentration that completely inhibited visible growth after 14 days of incubation at 37 °C. For quantitative analysis, viable counts from treated samples were compared with untreated controls, and survival rate (%) was calculated as (CFU/mL treated)/(CFU/mL control) × 100%. All experiments were performed in triplicate using independent cultures.

### 2.8. Bacterial Growth Curve

Bacterial growth kinetics were monitored in batch culture by measuring optical density at 600 nm (OD_600nm_), which correlates with cell density. The resulting growth curves reflected distinct phases of bacterial population physiology. For experimental treatments, cultures were grown to mid-log phase and exposed to either 0 µg/mL or 0.78 µg/mL ethionamide (ETH).

Bacterial suspensions were prepared in growth medium at an initial density of 1.2 × 10^6^ CFU/mL and incubated at 37 °C with orbital shaking (150 rpm). Growth was monitored at 0, 2, 4, 6, 8, 10, 12, 14, and 16 days by measuring OD_600nm_ in 96-well plates using a BioTek plate reader, with 100 μL aliquots collected at each time point. In these experiments, *Mtb* deletion mutants Δ137–176, Δ315–336, and Δ419–460 were generated as previously described [11,12,14,18,19], corresponding to disruptions in the respective EthA regions.

### 2.9. Expression Levels of Gene EthA Using RT-qPCR

*EthA* gene expression was quantified by real-time quantitative PCR (RT-qPCR). Briefly, 1 mL of late-log-phase bacterial culture (OD600 ≈ 1.0) was treated with either 0 μg/mL (untreated control) or 0.78 μg/mL ethionamide (ETH) for 16 days. Total RNA was extracted using the TransZol RNA Extraction Kit (Invitrogen, Carlsbad, CA, USA) according to the manufacturer’s instructions. RNA concentration and purity were assessed by spectrophotometry (A260/A280 ratio between 1.8 and 2.0), and RNA integrity was confirmed by agarose gel electrophoresis. First-strand cDNA was synthesized from 1 μg of total RNA using the PrimeScript 1st Strand cDNA Synthesis Kit (Takara, Kyoto, Japan) with random hexamer primers. RT-qPCR was performed using PowerUp SYBR Green Master Mix (Applied Biosystems, Foster city, CA, USA) with primers listed in Table S2, on an Applied Biosystems QuantStudio 5 system. Each reaction was performed in duplicate (technical replicates) for each biological replicate. The amplification efficiency for each primer pair was determined from standard curves and confirmed to be between 90% and 110%. Relative gene expression was calculated using the 2^−ΔΔCT^ method [50,51,52,53,54], with the untreated wild-type sample serving as the calibrator (ΔΔC*_T_* = [C*_T_*(*EthA*) − C*_T_*(*GyrA*)]_treated_ − [C*_T_*(*EthA*) − C*_T_*(*GyrA*)]_un-treated-WT_). Data are presented as log_2_-transformed fold changes in histogram format. Values above zero indicate upregulation, whereas values below zero indicate downregulation. The gyrase gene *GyrA* was used as the internal reference for normalization, and *GyrA* amplification served as a positive control. Three independent biological replicates were performed for each condition, and each biological replicate consisted of two technical replicates.

### 2.10. Accession Codes

The UniProt accession code for EthA (FAD-containing monooxygenase) from *Mycobacterium tuberculosis* (strain ATCC 25618/H37Rv) is P9WNF9.

### 2.11. Statistical Analysis

All experiments were performed with at least three independent biological replicates, with each biological replicate consisting of two to three technical replicates. Data are expressed as mean ± SD (standard deviation). Statistical analysis was performed using Origin 8.5, Microsoft Excel 2013, and SPSS 19.0. For comparisons involving more than two groups, one-way analysis of variance (ANOVA) was used. Prior to ANOVA, Bartlett’s test was applied to assess homogeneity of variances across groups; when variances were unequal, data were log-transformed prior to analysis. Following significant ANOVA results (*p* < 0.05), Tukey’s HSD (Honestly significant difference) post hoc test was performed for pairwise comparisons between groups. For comparisons between two groups, two-tailed Student’s t-test was used. In all statistical evaluations, *p* < 0.05 was considered statistically significant, and *p* < 0.01 was considered highly statistically significant.

## 3. Results

### 3.1. Hydrodynamic Properties of EthA in Solution

**Figure 2:**
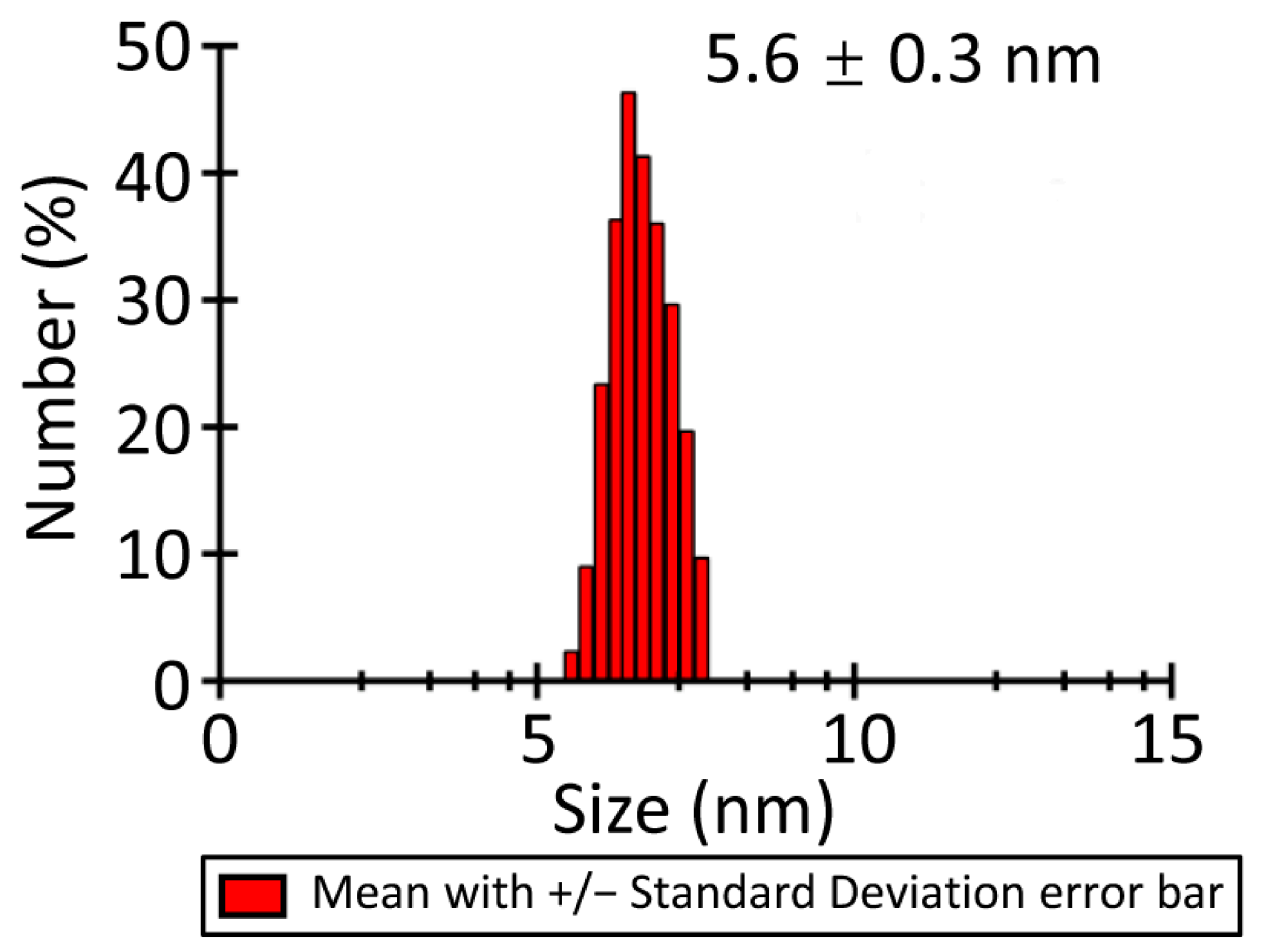
Dynamic light scattering (DLS) spectrum of EthA. The hydrodynamic diameter of EthA was measured to be 5.6 ± 0.3 nm (mean ± SD from three independent measurements).

Dynamic light scattering (DLS) was used to determine the hydrodynamic diameter of EthA to be 5.6 ± 0.3 nm (Figure 2 and Figure S5), which is consistent with a monomeric state.

To further investigate the monomer assignment, we estimated the theoretical minimum diameter of EthA using the empirical relation for anhydrous globular proteins: Dmin (nm) = 0.132 × M^1/3^ (M in Da) [35]. For a monomeric molecular weight of 55,326 Da, this yields Dmin ≈ 5.0 nm. Our measured hydrodynamic diameter of 5.6 nm exceeds this predicted minimum by ~12%, which is within the 10–20% range typically expected for a hydrated monomer. In contrast, the corresponding minimum diameter for a dimer would be 6.3 nm, substantially larger than our experimental value. Moreover, the post-centrifugation DLS intensity distribution exhibited a single narrow peak (Figure 2; Table S3), confirming sample monodispersity. Collectively, these data are consistent with EthA existing predominantly as a monomer under the conditions tested. However, as DLS measures hydrodynamic size rather than molecular mass directly, we cannot entirely exclude the presence of transient dimers or higher-order species that might not be resolved under these conditions.

### 3.2. Structural Model and Quality Assessment of EthA

**Figure 3:**
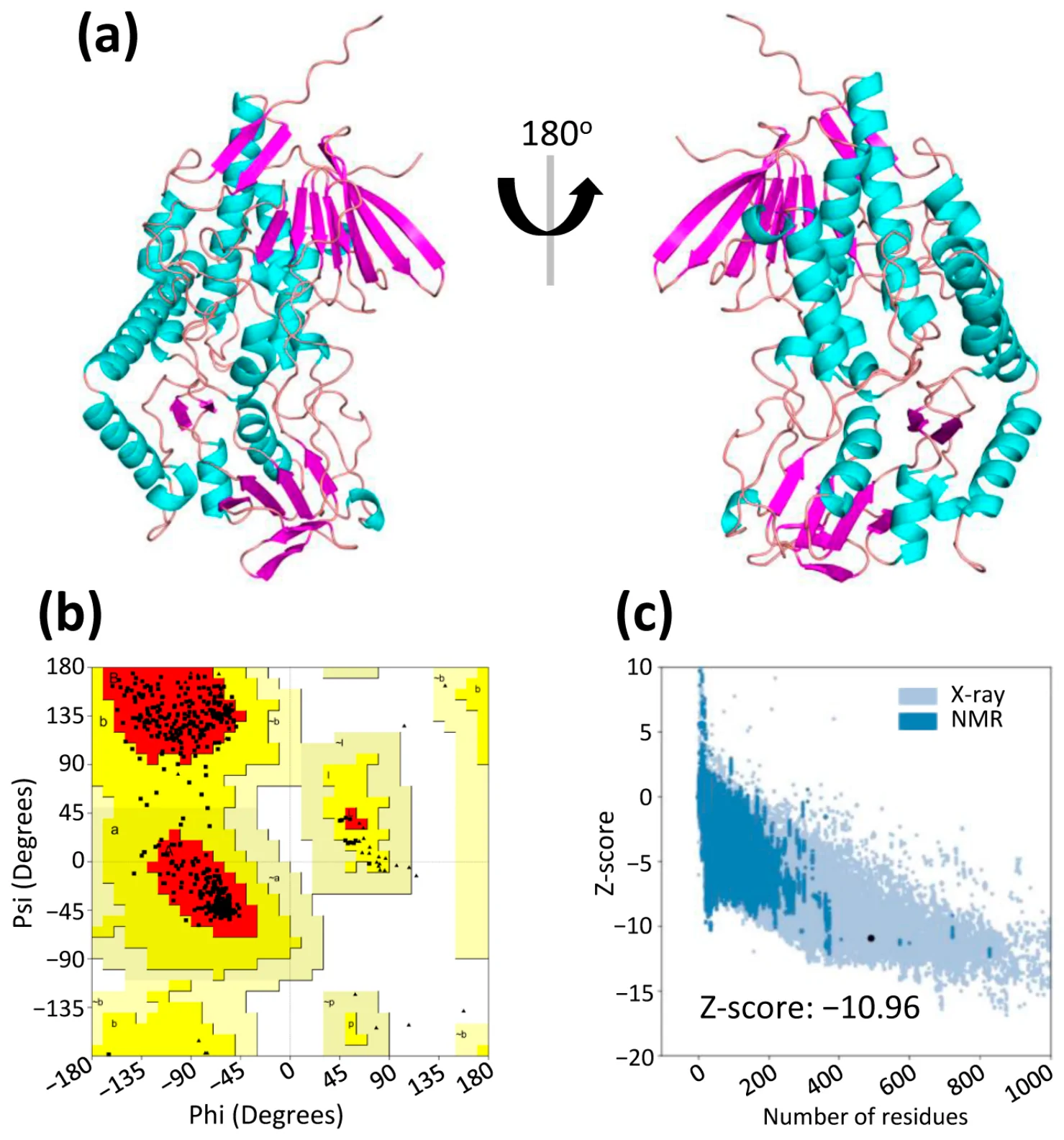
Structural modeling and quality evaluation of EthA. (**a**) The structural model of EthA, predicted using AlphaFold2, is presented as cyan ribbon diagrams from two distinct viewpoints, with alpha-helices in pink and beta-sheets in cyan. (**b**) Model validation via Ramachandran plot analysis shows residues in the most favored conformations in red, whereas those in progressively less favored regions are depicted in lighter shades. (**c**) ProSA analysis yields a Z-score of −10.96, confirming the high reliability of the predicted model.

The three-dimensional structure of EthA was predicted using AlphaFold2 (Figure 3a and Figure S6). The resulting model adopts a canonical fold characteristic of the FAD-dependent monooxygenase family, featuring a central beta-sheet core flanked by α-helices on both sides. The overall architecture displays a bipartite organization, with the N-terminal domain forming the FAD-binding site and the C-terminal domain contributing to NADPH binding and substrate recognition.

Model validation was performed using Ramachandran plot analysis (Figure 3b; Table 1). A total of 387 residues (91.7%) were located in the most favored regions, while 35 residues (8.3%) were present in additionally allowed regions; no residues were found in generously allowed or disallowed regions. Glycine and proline residues (39 and 26, respectively), as well as two terminal residues, were excluded from the analysis. These results indicate that the backbone dihedral angles are geometrically well defined.

To further assess the local confidence of the predicted model, we examined the AlphaFold2 pLDDT (predicted Local Distance Difference Test) scores. The average pLDDT for the EthA model was 94.81, indicating high confidence for the majority of the structure. Notably, the three peripheral segments of interest (residues 137–176, 315–336, and 419–460) showed pLDDT values of 92.3, 98.5, and 75.1, respectively (Figure S5), suggesting moderate-to-high confidence. The PAE (Predicted aligned error) plots (Figure S6) showed low error values for the core FAD- and NADPH-binding domains, while higher error values were observed for the peripheral regions, further corroborating their increased conformational variability. Overall, the confidence metrics indicate that the predicted positions of the core functional domains are highly reliable, whereas the peripheral segments, while predicted with moderate confidence, are appropriately characterized as flexible regions.

Overall model quality was further assessed using the ProSA web server (Figure 3c). The resulting Z-score of −10.96 falls within the range typically observed for experimentally determined structures of comparable size, supporting the reliability and global accuracy of the AlphaFold2-predicted EthA model.

### 3.3. Binding Mode of NADPH in the EthA Binary Complex

**Figure 4:**
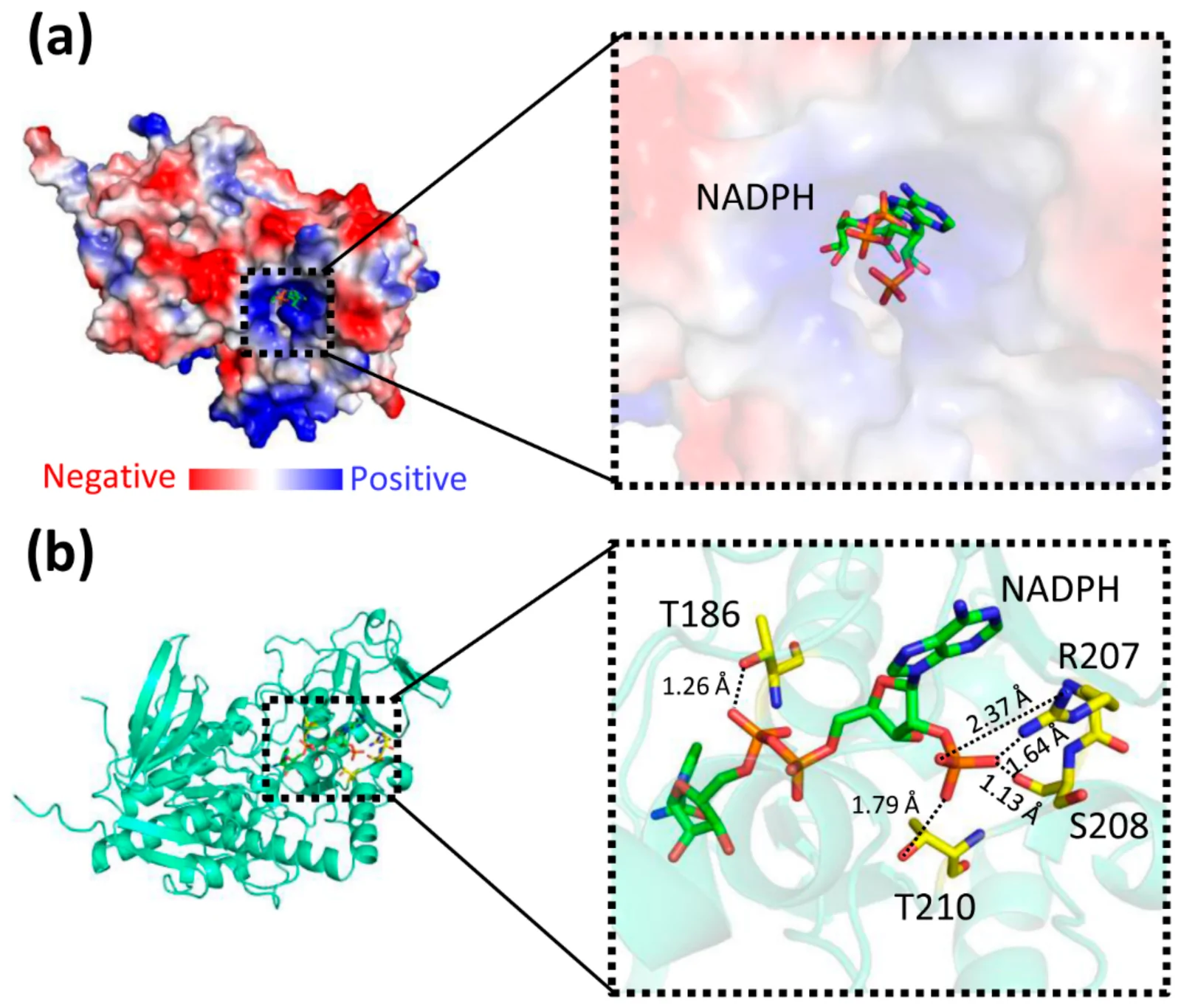
Model of the EthA-NADPH binary complex. NADPH (nicotinamide adenine dinucleotide phosphate) is shown in stick representation. (**a**) Surface representation of the model, colored by electrostatic potential: red for negative, blue for positive, and white for neutral. (**b**) Ribbon representation of the model, with a magnified view of the binding sites on the right. Dashed lines indicate polar and charged interactions.

In the EthA-NADPH binary complex obtained from docking, electrostatic surface representation revealed that the NADPH-binding pocket is predominantly positively charged, providing electrostatic complementarity to the negatively charged phosphate groups of NADPH (Figure 4a). The binding cavity forms a surface cleft that accommodates NADPH in an extended conformation. It is important to note that this docking analysis focused on NADPH binding rather than ethionamide recognition, as the structural basis for NADPH binding is better understood from related monooxygenases and provides a reliable reference for validating the docking approach. The ethionamide binding mode was not directly modeled in this study, as our primary interest was to use the structural model to guide mutagenesis of active-site residues.

Structural analysis of the EthA-NADPH interactions showed that the nicotinamide ring is positioned in close proximity to the FAD-binding region (Figure 4b), consistent with a catalytically competent hydride transfer geometry. The 2′-phosphate group of the adenosine ribose forms polar interactions with basic residues, while the pyrophosphate moiety engages in multiple hydrogen bonds with backbone amides and side-chain hydroxyl groups. The adenine ring is stabilized within a hydrophobic sub-pocket, and the nicotinamide moiety is oriented toward the catalytic center, with the C_4_ atom positioned optimally for potential hydride transfer to FAD.

### 3.4. Effects of Active-Site Mutations on EthA Catalytic Activity

**Figure 5:**
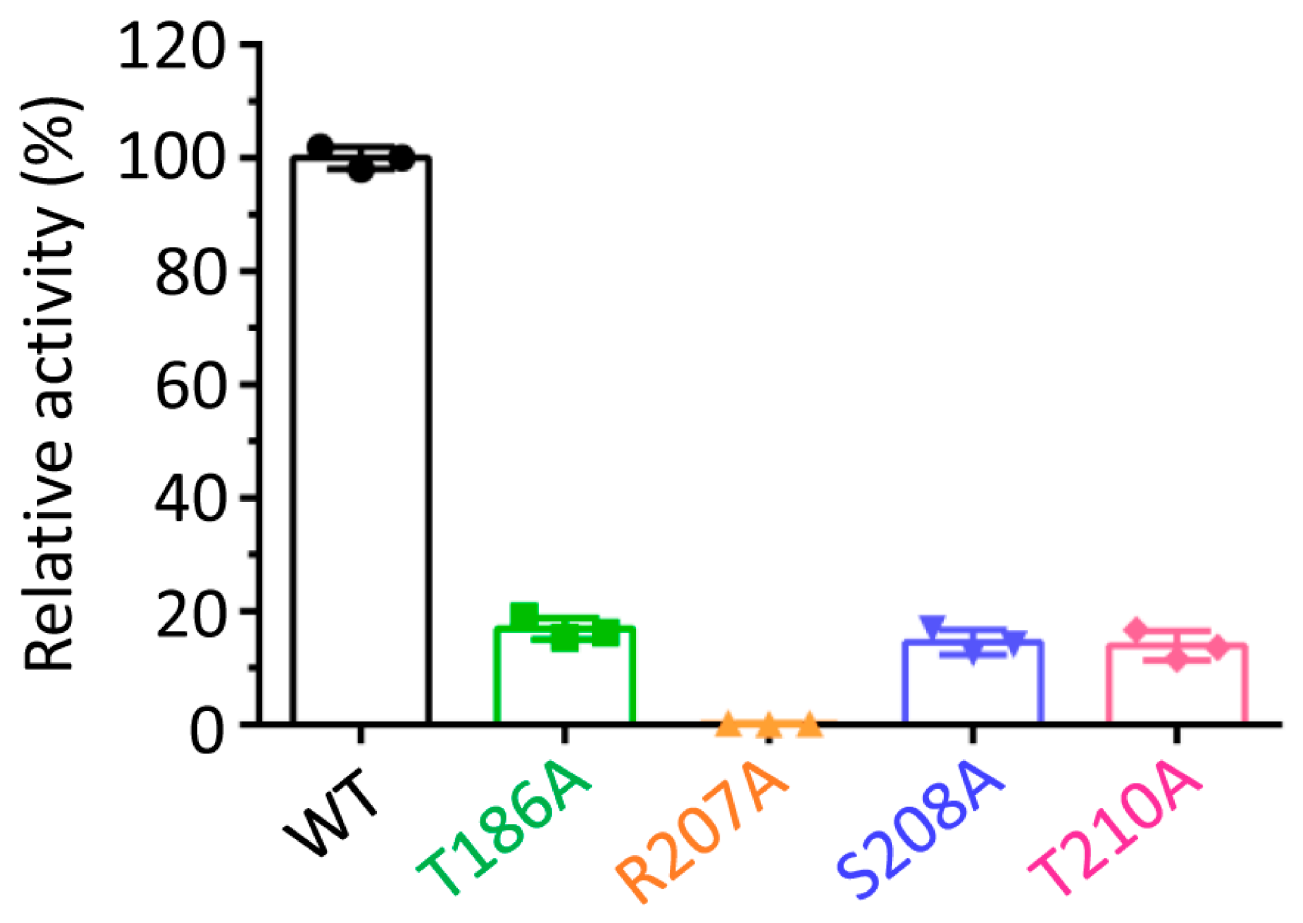
Enzymatic characterization of EthA. The T186A, S208A and T210A mutants exhibited 5.8- to 7.2-fold decreases in activity, whereas the R207A mutant led to complete loss of activity. The activity of WT (wild-type) protein was set to 100%. The positive control substrate phenylacetone (250 μM) yielded a specific activity of 8.1 nmol NADPH consumed/min/nmol FAD (mean ± SD, *n* = 3), confirming the functional integrity of the enzyme preparation and assay conditions. Data are presented as mean ± SD from three independent biological replicates (*n* = 3). Compared to the wild type (WT) (one-way ANOVA followed by Tukey’s HSD post hoc test), exact *p*-values are as follows: T186A: *p* = 0.0012, R207A: *p* < 0.0001, S208A: *p* = 0.0008, and T210A: *p* = 0.0015.

The enzymatic activities of wild-type EthA and four site-directed mutants (T186A, S208A, R207A, and T210A) were evaluated under identical assay conditions (Figure 5). To validate the assay methodology, phenylacetone, a known substrate of Baeyer–Villiger monooxygenases, was used as a positive control. This yielded a specific activity of 8.1 nmol of NADPH consumed per minute per nmol of FAD. This result confirmed the functional integrity of the enzyme preparation and the reliability of the assay conditions. The T186A mutant retained 16.9% of wild-type (WT) activity, while the S208A variant exhibited a 6.9-fold reduction in activity relative to WT protein. Similarly, the T210A mutant showed reduced activity, retaining 13.9% of WT activity. In contrast, the R207A mutant completely abolished detectable enzymatic activity. These results indicate that T186, S208, and T210 contribute to enzymatic activity but are not essential for activity, whereas R207 is indispensable for EthA function.

### 3.5. Modulation of EthA Activity by Peripheral Regions of the Catalytic Cavity

**Figure 6:**
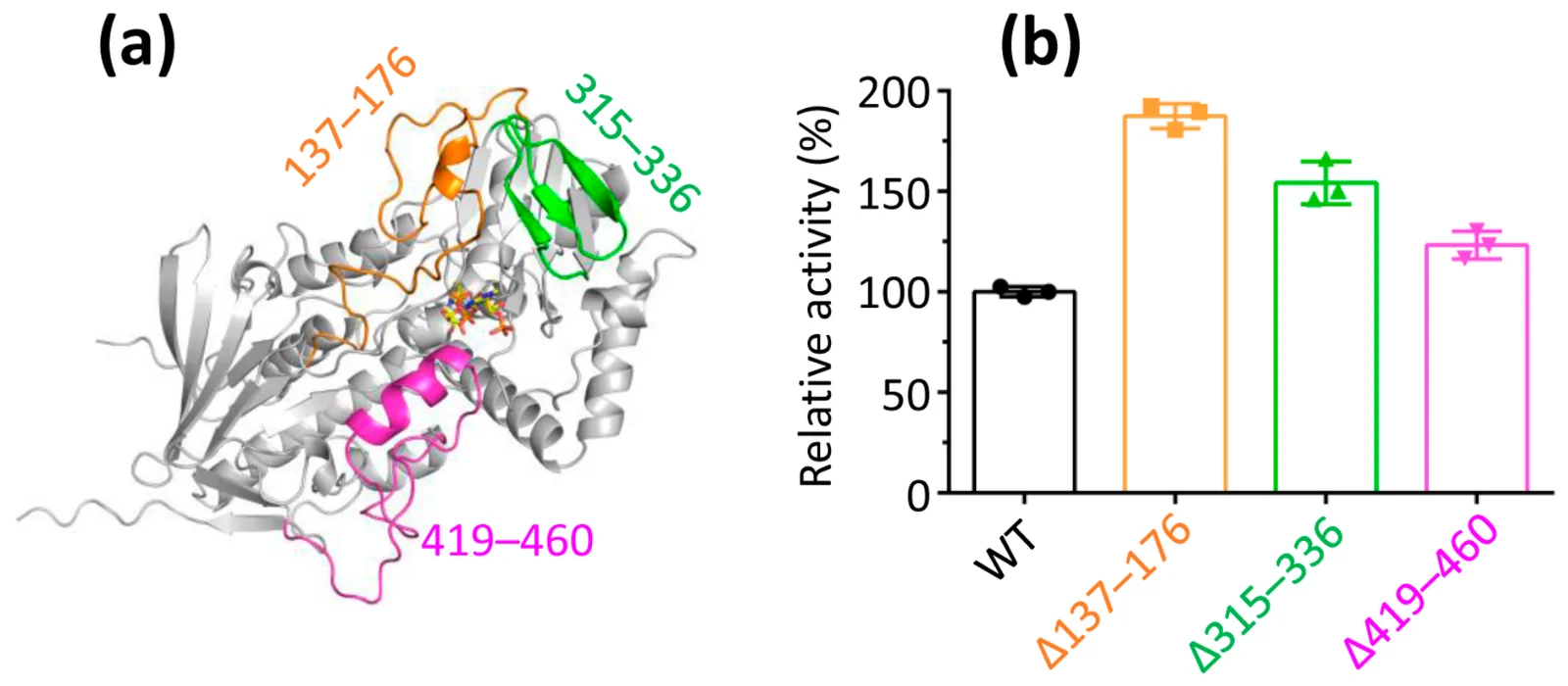
Regulation of EthA activity by peripheral regions of the catalytic cavity. (**a**) Residue regions 137–176, 315–336 and 419–460 were colored orange, green and magenta, respectively. (**b**) Deletion of segments 137–176, 315–336 and 419–460 (∆137–176, ∆315–336 and ∆419–460) increased the activity 1.9-fold, 1.5-fold, and 1.2-fold, respectively. The activity of WT (wild-type) protein was set to 100%. Data are presented as mean ± SD from three independent biological replicates (*n* = 3). Compared to the wild type (WT) (one-way ANOVA followed by Tukey’s HSD post hoc test), exact *p*-values: Δ137–176, *p* = 0.0023; Δ315–336, *p* = 0.0156; Δ419–460, *p* = 0.0342.

We investigated the role of three peripheral regions of the catalytic cavity in regulating EthA enzymatic activity. These segments (137–176, 315–336, and 419–460) are located at the periphery of the catalytic pocket (Figure 6a) and do not directly participate in cofactor binding or the catalytic center.

Deletion of region 137–176 (Δ137–176) resulted in a 1.9-fold increase in activity relative to the wild-type protein (Figure 6b). Similarly, deletion of 315–336 (Δ315–336) increased activity by 1.5-fold, while deleting 419–460 (Δ419–460) produced a more modest 1.2-fold enhancement (Figure 6b). Collectively, all three deletion mutants exhibited elevated activity, indicating that these peripheral regions exert a negative regulatory effect on EthA catalysis. The magnitude of this effect varied among segments, with region 137–176 showing the strongest influence on enzymatic activity.

### 3.6. Backbone Flexibility of EthA Peripheral Regions

**Figure 7:**
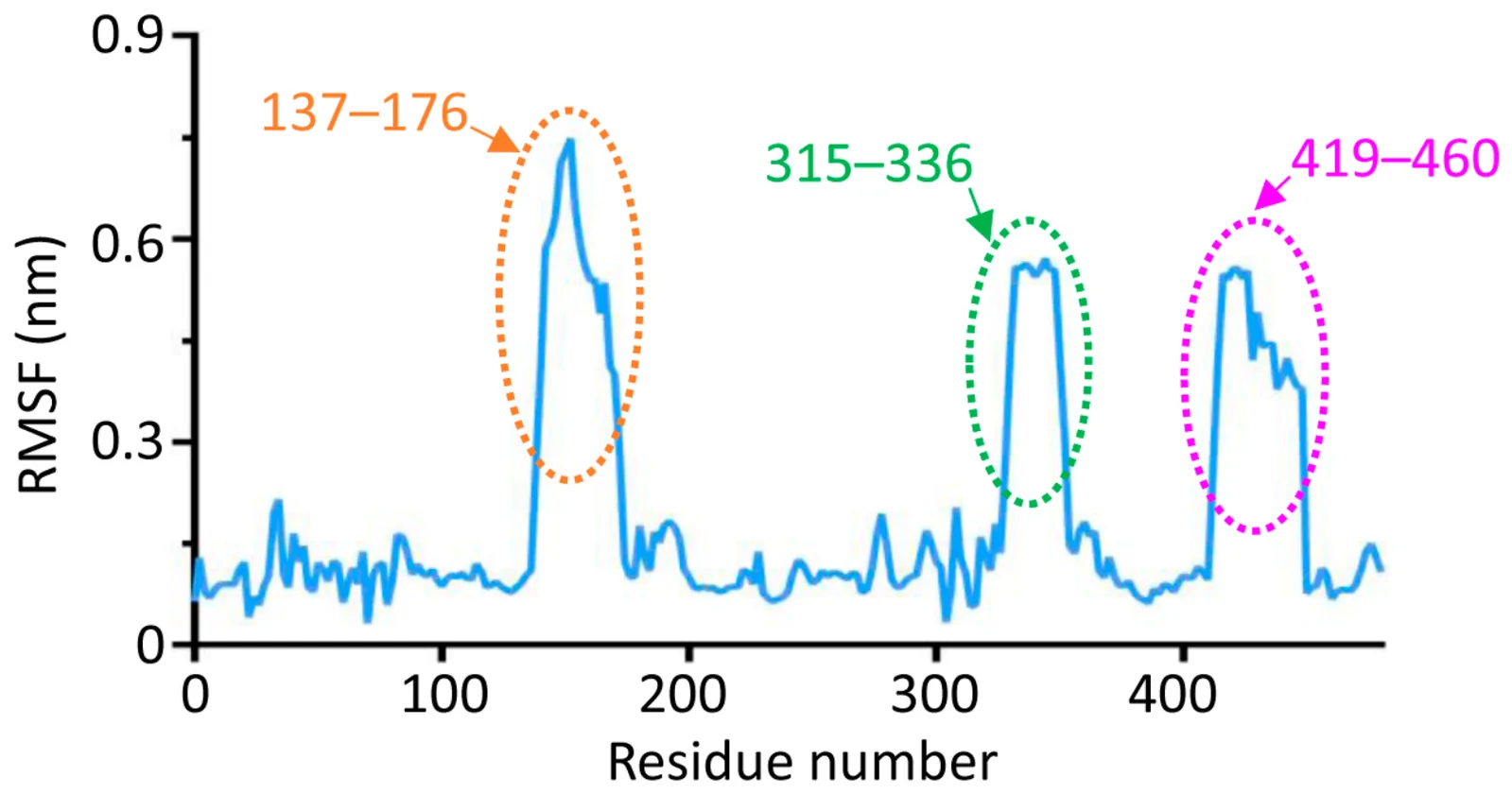
The high flexibility of residues 137–176, 315–336 and 419–460 based on the root mean square fluctuation (RMSF) profile of EthA. Elevated root-mean-square fluctuation (RMSF) peaks in segments 137–176, 315–336, and 419–460 indicate markedly higher backbone flexibility in these regions relative to the rest of EthA. RMSF quantifies residue flexibility by measuring the time-averaged deviation of backbone atoms from their reference positions, reflecting their relative fluctuations over time.

The root-mean-square fluctuation (RMSF) values quantify time-averaged deviations of backbone atoms from their reference positions and provide a measure of residue-specific flexibility across the protein (Figure 7). Pronounced RMSF peaks were observed in three regions (residues 137–176, 315–336, and 419–460), indicating substantially increased local flexibility relative to the rest of the structure. In contrast, the core structural elements, including the beta-sheet and alpha-helical regions that constitute the FAD- and NADPH-binding domains, exhibited low RMSF values, consistent with their rigid and well-ordered nature. Notably, the highly flexible segments identified by RMSF analysis correspond to the peripheral regions implicated in the deletion studies (Figure 6b).

### 3.7. EthA Expression and Phenotypic Effects in Response to Ethionamide Treatment

The susceptibility of peripheral-region deletion mutants to ethionamide (ETH) was assessed by minimum inhibitory concentration (MIC) measurements (Table 2). The MIC of ETH for the wild-type (WT) strain was 3.13 µg/mL, whereas the Δ137–176, Δ315–336, and Δ419–460 mutants exhibited MIC values of 0.78, 1.56, and 1.56 µg/mL, respectively, indicating increased ETH susceptibility. The validity of the MIC assay conditions was confirmed by testing the positive control drugs isoniazid (INH) and rifampicin (RIF), which produced MIC values of 0.05 and 0.10 μg/mL, respectively. The MIC ranking (Δ137–176 < Δ315–336 ≈ Δ419–460 < WT) was consistent with the relative enzymatic activities observed previously (Figure 6), in which Δ137–176 showed the greatest activity enhancement (1.9-fold), followed by Δ315–336 (1.5-fold) and Δ419–460 (1.2-fold).

**Figure 8:**
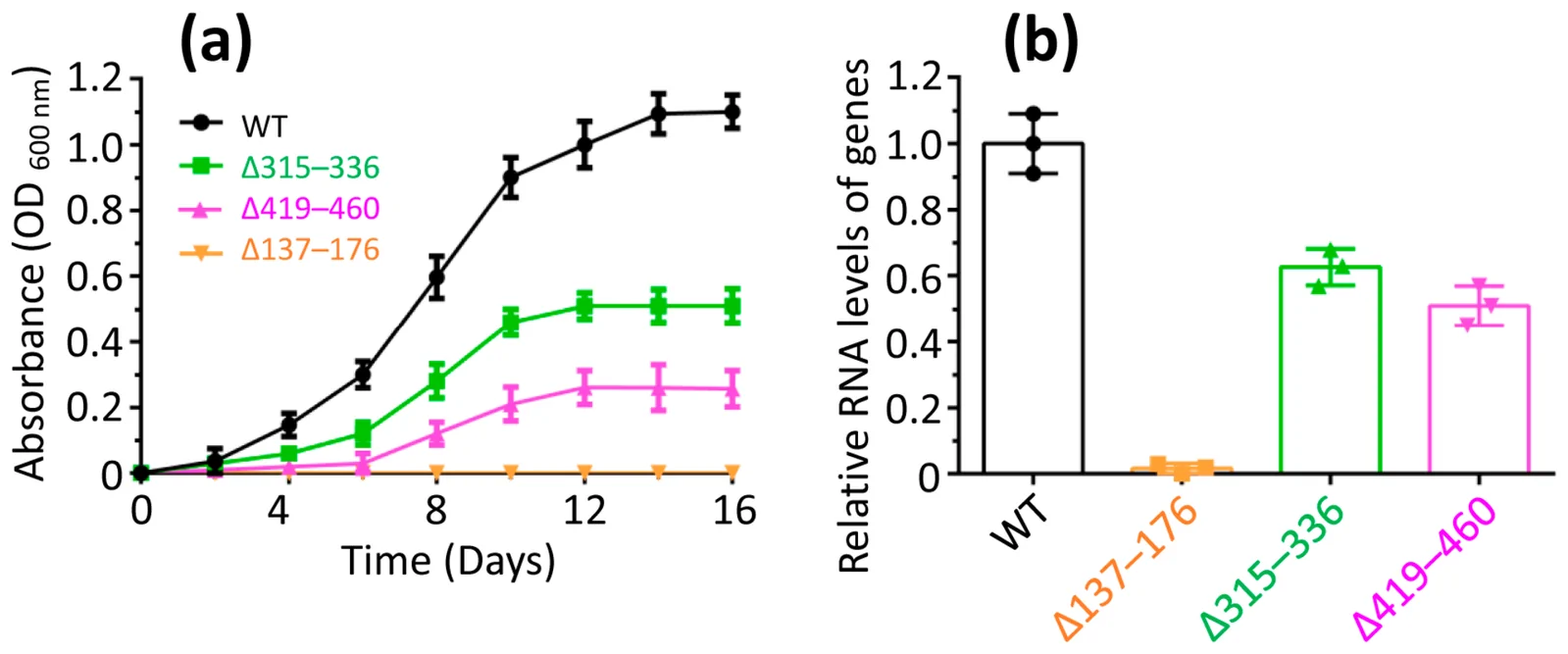
Functional role of EthA. (**a**) Growth curves of bacterial strains following treatment with 0.78 µg/mL ethionamide (ETH). Data represent mean ± SD from three independent biological replicates (*n* = 3). (**b**) Expression levels of *EthA* gene under the same treatment condition (0.78 µg/mL ETH), measured by RT-qPCR and presented as log_2_-fold changes relative to untreated wild-type (WT) control (calibrator). The log_2_-fold change was calculated as log_2_(2^−ΔΔCT^), where values above 0 indicate upregulation and values below 0 indicate downregulation relative to the calibrator. Baseline expression in wild-type (WT) strains was set to “1”. Data are presented as mean ± SD from three independent biological replicates (*n* = 3). *p* < 0.05, *p* < 0.01 versus WT-treated (one-way ANOVA followed by Tukey’s HSD post hoc test).

Consistently, the WT strain displayed a marked reduction in growth upon exposure to ETH (0.78 µg/mL), with growth inhibition evident within the first 12 days. In contrast, strains with impaired EthA function exhibited attenuated growth inhibition under the same conditions, supporting a correlation between EthA activity and ETH susceptibility (Figure 8a).

EthA transcript levels under ETH treatment (0.78 µg/mL) were further quantified by RT-qPCR and expressed as log_2_ fold changes relative to untreated controls (Figure 8b). ETH exposure induced EthA expression in all strains, as indicated by positive log_2_ fold changes, with the strongest induction observed in strains retaining functional EthA. These results indicate that EthA expression is responsive to ETH treatment and that the magnitude of induction is associated with EthA functional status.

## 4. Discussion

In this work, we used AlphaFold2 to generate a structural model of EthA, which was validated using Ramachandran statistics (91.7% of residues in favored regions) and a ProSA Z-score of −10.96. Molecular docking analysis was performed to guide functional interpretation. Site-directed mutagenesis revealed that R207 is essential for enzymatic activity, whereas T186A, S208A, or T210A reduced activity by 5.8- to 7.2-fold. In contrast, deletion of peripheral segments 137–176, 315–336, and 419–460 increased activity by 1.9-, 1.5-, and 1.2-fold, respectively. These regions also exhibited elevated backbone flexibility in molecular dynamics simulations. Consistently, the deletion mutants showed increased ethionamide susceptibility, with MIC values decreasing from 3.13 µg/mL in the wild type to 0.78 µg/mL in Δ137–176. Together, these findings establish a structural framework for EthA function and suggest that flexible peripheral regions may negatively regulate catalytic activity.

**Figure 9:**
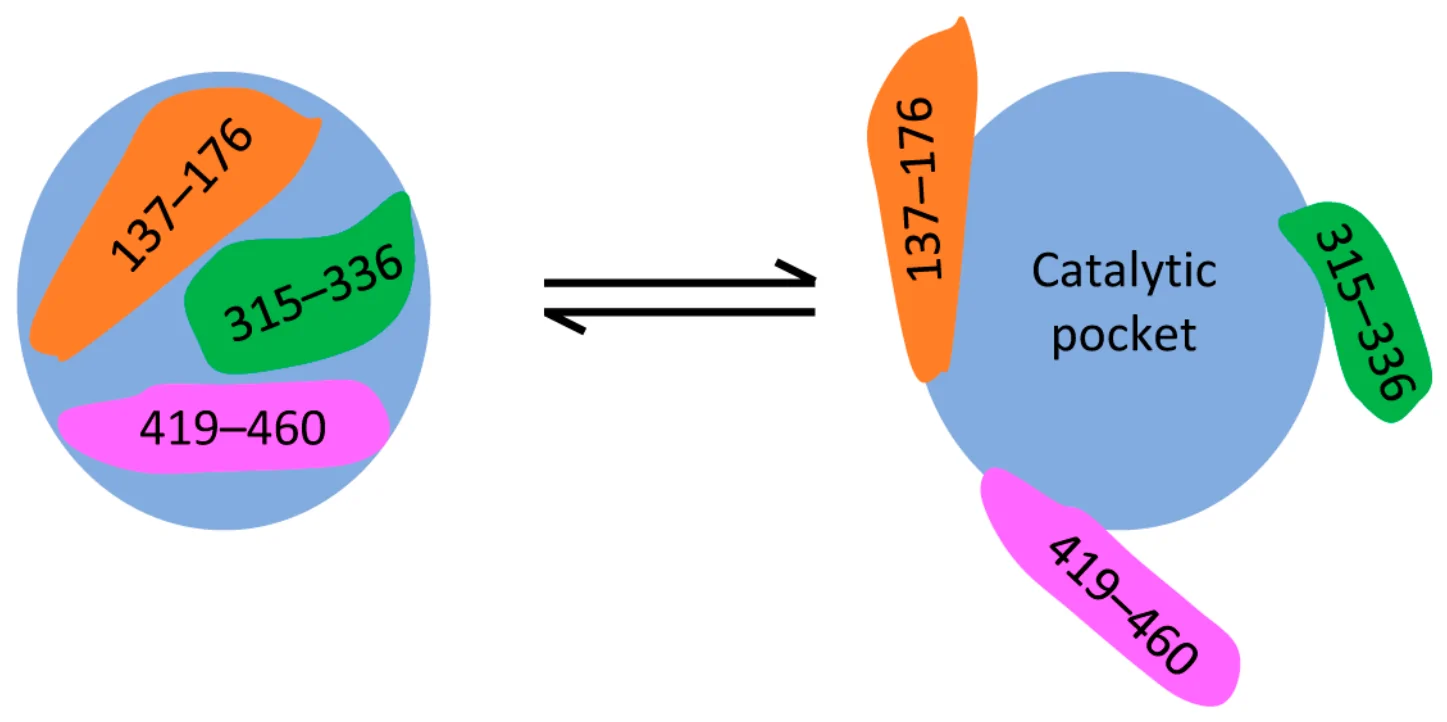
The flexibility of segments 137–176, 315–336, and 419–460 modulates EthA catalytic activity. Rearrangement of regions 137–176 (orange), 315–336 (green), and 419–460 (magenta) enlarges the catalytic pocket, which enhances substrate binding and thereby activates the catalytic activity of EthA.

Based on the structural and functional findings, we propose a schematic model describing the conformational dynamics of three flexible segments (137–176, 315–336, and 419–460) and their impact on EthA catalytic activity (Figure 9). The model suggests a transition from a more restricted to a more open conformation upon rearrangement of these peripheral regions. This structural shift leads to an expansion of the catalytic pocket, as reflected by increased cavity volume in the model, which is proposed to enhance substrate accessibility and thereby promote catalytic turnover. The extent of pocket expansion correlates with the observed activity changes among the deletion mutants. Specifically, the Δ137–176 mutant, which exhibited the greatest increase in activity, is associated with the most pronounced conformational opening, whereas the Δ315–336 and Δ419–460 mutants show progressively smaller structural expansions consistent with their more modest activity enhancements.

The mechanistic link between increased backbone flexibility in the peripheral regions and enhanced enzymatic activity can be understood through several interrelated factors. First, these flexible segments may act as conformational ‘brakes’ that restrict the mobility of the catalytic pocket; their removal allows for greater sampling of catalytically competent conformations. Second, the increased flexibility may facilitate substrate access by reducing steric hindrance at the entrance to the active site, as supported by the observed expansion of the catalytic pocket in our structural model (Figure 9). Third, local conformational dynamics may influence the positioning of key catalytic residues relative to FAD and/or NADPH, thereby modulating hydride transfer efficiency. Notably, this proposed regulatory mechanism, whereby peripheral loops constrain the activity of a FAD-dependent monooxygenase, finds support in studies of related Baeyer–Villiger monooxygenases, in which loop dynamics have been shown to influence substrate specificity and catalytic turnover [55,56].

Our findings also have implications for understanding ethionamide resistance mechanisms. Previous genomic analyses of clinical *Mtb* isolates have identified numerous EthA mutations associated with ethionamide resistance, including missense, nonsense, and frameshift variants [12,18]. Many of these resistance-conferring mutations cluster in the FAD- and NADPH-binding domains, consistent with their essential roles in catalytic function. However, mutations in peripheral regions have been less frequently reported, possibly because they may only moderately affect activity or because they are not detected as primary resistance determinants in clinical screens. Interestingly, the peripheral regions we identified (137–176, 315–336, and 419–460) are located in surface-exposed loops that are not directly involved in cofactor binding. Mutations in these regions might confer partial resistance by modestly reducing EthA activity, potentially in combination with other mutations that together lead to high-level resistance [10]. Our finding that deletion of these regions actually increases activity raises the possibility that gain-of-function mutations in EthA could paradoxically increase ethionamide sensitivity, a phenomenon that might be exploited therapeutically [6]. These observations underscore the complexity of ethionamide resistance mechanisms and suggest that the peripheral regions of EthA could represent previously unrecognized targets for modulating drug activation.

The MIC measurements provide supporting evidence linking EthA activity to whole-cell ethionamide susceptibility. The decreased MIC values observed for the deletion mutants (Δ137–176, Δ315–336, and Δ419–460) correlate well with their increased enzymatic activities (Figure 6), suggesting that enhanced EthA activity results in greater prodrug activation and consequently increased ethionamide sensitivity. However, several caveats should be noted. First, whole-cell ethionamide susceptibility is a complex phenotype influenced by multiple factors beyond EthA activity, including drug uptake and efflux [57], cell wall permeability [58], and the activity of other enzymes that may contribute to ethionamide metabolism [59]. Second, *EthA* expression is regulated at the transcriptional level by the repressor EthR [11], and changes in EthA protein levels under different growth conditions could affect susceptibility independently of catalytic activity. Third, mutations in other genes, such as *InhA* or *Ndh*, can confer ethionamide resistance without affecting EthA function [10]. Therefore, while the MIC data support the biochemical findings, they should be interpreted as correlative evidence rather than direct proof of the catalytic mechanism. Future studies using isogenic strains and controlled expression systems would help further dissect the contribution of EthA activity to ethionamide susceptibility.

Several limitations of this study should be acknowledged. First, the structural model is derived from AlphaFold2 predictions rather than experimentally determined structures (e.g., X-ray crystallography or cryo-EM). Although validation metrics indicate high reliability, subtle conformational features and ligand-induced structural changes may not be fully captured. Second, the deleted segments (137–176, 315–336, and 419–460) are relatively large (40, 22, and 42 residues, respectively), raising the possibility that the observed activity increases could result from local structural perturbations beyond the intended deletions, such as altered packing interactions, changes in secondary structure, or indirect effects on the catalytic pocket geometry. We cannot exclude the possibility of localized conformational rearrangements that might affect substrate accessibility or cofactor binding. Nevertheless, the strong correlation between the magnitude of activity enhancement and RMSF values across the three regions (Figure 7) supports the interpretation that local flexibility is a key determinant of activity, though not the sole factor. Third, enzymatic assays and MIC measurements were conducted under standard in vitro conditions, which may not fully reflect the physiological environment within host macrophages. Future in vivo studies will be required to validate the biological relevance of these findings.

Our DLS analyses establish that all mutants remain monodisperse, with hydrodynamic radii closely matching those of the wild-type protein, and critically, no aggregation is detected under our experimental conditions. This observation robustly excludes the possibility of gross hydrophobic collapse or nonspecific oligomerization. Nevertheless, we acknowledge that direct spectroscopic verification of secondary structure retention is not provided in this study, as far-UV CD (Circular dichroism) spectroscopy was precluded by severe buffer incompatibility; our mandatory assay buffer contains 50 mM Tris-HCl, 150 mM NaCl, and 10% glycerol, all of which collectively cause dominant far-UV absorption and high-conductivity artifacts that would compromise spectral reliability. Importantly, however, the major confounding factor in CD-based structural inference, namely light scattering arising from protein aggregates, has been effectively eliminated by our DLS data. Therefore, while we cannot make absolute claims regarding unchanged global α-helix/β-sheet percentages, we can confidently conclude that the mutations do not perturb the native quaternary assembly or overall compactness. Taken together with the above results, the observed functional differences are more plausibly attributed to localized alterations in the active-site microenvironment or backbone dynamics, rather than to large-scale conformational rearrangements.

Additionally, our DLS-based assignment of EthA as a monomer should be interpreted with caution, as DLS measures hydrodynamic size rather than molecular mass directly (Figure S7; Table S3). Future studies using size-exclusion chromatography coupled with multi-angle light scattering (SEC-MALS) or analytical ultracentrifugation would provide more definitive information about the oligomeric state of EthA.

In conclusion, we present a structural and functional characterization of EthA, identifying key catalytic residues and providing evidence that flexible peripheral regions are capable of modulating enzymatic activity. This study advances the molecular understanding of ethionamide activation and provides a framework for structure-based approaches to overcome ethionamide resistance, including the design of EthA activators or the targeting of inhibitory loop regions to enhance prodrug activation.

## 5. Conclusions

In this study, we provide a structural and functional characterization of EthA, the FAD-containing monooxygenase responsible for activating the antitubercular prodrug ethionamide in *Mtb*. Using an AlphaFold2-predicted structural model validated by Ramachandran statistics and ProSA analysis, we identified key catalytic residues and peripheral regions that modulate enzymatic activity. Site-directed mutagenesis revealed that R207 is required for detectable EthA activity, while alanine substitutions at T186, S208, and T210 result in 5.8- to 7.2-fold reductions in activity. Notably, deletion of three flexible peripheral segments (residues 137–176, 315–336, and 419–460) enhances EthA activity by up to 1.9-fold, correlating with increased ethionamide susceptibility as reflected by reduced MIC values. Molecular dynamics simulations indicate that these regions exhibit elevated backbone flexibility, supporting a model in which peripheral loop dynamics constrain catalytic function. These findings establish a structural framework for EthA-mediated ethionamide activation and suggest that modulation of peripheral loop flexibility represents a previously unrecognized regulatory mechanism. This work advances the molecular understanding of a key drug-activating enzyme in *Mtb* and may inform structure-based strategies to enhance ethionamide efficacy or overcome resistance in clinical settings.

## Figures and Tables

**Table 1:** Ramachandran plot statistics of structural models of EthA.

Residual Properties	Number of Residues	Total % of Residues
Most favored region	387	91.7
Additional allowed region	35	8.3
Generously allowed region	0	0
Disallowed region	0	0

Note: Number of end residues (excl. Gly and Pro): 2; Number of glycine residues (shown as triangles): 39; Number of proline residues: 26.

**Table 2:** Susceptibility of *Mtb* to ETH (Ethionamide).

Strains	WT	Δ137–176	Δ315–336	Δ419–460
MIC *^a^* (µg/mL)	3.13	0.78	1.56	1.56

*^a^* The MIC (Minimum inhibitory concentration) is the lowest concentration of antibacterial materials that results in no observable growth. INH (isoniazid) and RIF (rifampicin) were used as positive controls for the MIC assay. Values for INH and RIF represent mean ± SD from three independent experiments, confirming the validity of our experimental conditions. The MIC values for INH and RIF were 0.05 and 0.10 μg/mL, respectively.

## Data Availability

The data presented in this study are available in this article (and Supplementary Materials). Further inquiries can be directed to the corresponding author.

## Supplementary Materials

The following supporting information can be downloaded at: https://www.mdpi.com/article/10.3390/biom16091333/s1, Figure S1: The amino acid sequence of EthA (FAD-containing monooxygenase, UniProt code P9WNF9); Figure S2: The optimized sequence of gene EthA; Figure S3: The chemical structure of NADPH (Nicotinamide adenine dinucleotide phosphate); Figure S4: The chemical structure of ETH (Ethionamide); Figure S5: The confidence of the AlphaFold2-predicted EthA structure was evaluated using the per-residue Local Distance Difference Test (pLDDT), a 0–100 scale wherein higher scores denote greater predicted local accuracy; Figure S6: Predicted aligned error (PAE); Figure S7: Dynamic light scattering (DLS) analysis of different EthA mutants; Table S1: Primers used for generating site-directed mutants of EthA; Table S2: Primers used for RT-qPCR; Table S3: Dynamic light scattering parameters of purified EthA.
