# Supplementary material for: The Flexible Peripheral Segments Modulate the Catalytic Activity of the FAD-Containing Monooxygenase EthA from Mycobacterium tuberculosis
Source: Biomolecules. 2026 Sep 14;16(9):1333. doi: 10.3390/biom16091333 (PMC13604814; doi:10.3390/biom16091333)
Supplement: Supplementary file 1 [file biomolecules-16-01333-s001.zip › biomolecules-4540043-supplementary.pdf]

Supplementary Material

The flexible peripheral segments modulate the catalytic activity of the FAD-containing monooxygenase EthA from *Mycobacterium tuberculosis*

Dafeng Liu[1,2,*], Xieping Sun[1], Feng Yu[1], Hongying Song[2], Wenshuang Yao[2], Huashui Deng[2] and Daoqi Song[2]

[1]College of Biological Sciences and Technology, Yili Normal University, Yining 835000, Xinjiang, China;

[2]School of Life Sciences, Xiamen University, Xiamen 361102, Fujian, China.

[*]Correspondence: dafeli@sina.cn or liudafeng2017@163.com

Figure S1.

MTEHLDVVIVGAGISGVSAAWHLQDRCPTKSYAILEKRESMGGTWDLFRYPGIRSDSDMYTLGFRFRPWTGR
QAIADGKPILEYVKSTAAMYGIDRHIRFHHKVISADWSTAENRWTVHIQSHGTLSALTCEFLFLCSGYYNYDEGY
SPRFAGSEDFVGPIIHPQHWPEDLDYDAKNIVVIGSGATAVTLVPALADSGAKHVTMLQRSPTYIVSQPDRDGIA
EKLNRWLPETMAYTAVRWKNVLRQAAVYSACQKWPRRMRKMFLSLIQRQLPEGYDVRKHFGPHYNPWDQ
RLCLVPNGDLFRAIRHGKVEVVTDTIERFTATGIRLNSGRELPADIIITATGLNLQLFGGATATIDGQQVDITTTMAY
KGMMLSGIPNMAYTVGYTNASWTLKADLVSEFVCRLLNYMDDNGFDTVVVERPGSDVEERPFMEFTPGYVL
RSLDELPKQGSRTPWRLNQNYLRDIRLIRRGKIDDEGLRFAKRPAPVGV

Figure S1. The amino acid sequence of EthA (FAD-containing monooxygenase, UniProt code P9WNF9).

Figure S2.

ATGACCGAACATCTGGACGTTGTTATTGTAGGTGCTGGTATCAGCGGTGTCTCTGCTGCCTGGCATCTGCAG
GATCGTTGCCCGACTAAAAGCTACGCGATCCTGGAAAAACGTGAATCCATGGGTGGCACCTGGGACCTGTT
TCGTTACCCAGGCATCCGCTCCGATTCCGACATGTACACTCTGGGTTTCCGCTTCCGTCCTTGGACCGGCCGC
CAGGCAATCGCGGATGGTAAACCAATCCTGGAGTACGTGAAATCTACGGCAGCGATGTACGGCATCGACCG
CCACATTCGTTTCCACCACAAAGTTATCTCCGCGGACTGGTCTACGGCTGAGAACCGTTGGACGGTTCATATT
CAGTCTCATGGTACCCTGTCTGCACTGACCTGCGAATTCCTGTTCCTGTGCTCTGGTTACTACAACTACGACG
AAGGTTACTCTCCTCGTTTCGCTGGCTCTGAAGACTTCGTAGGCCCGATCATCCACCCGCAGCACTGGCCAG
AAGATCTGGACTACGACGCAAAGAACATCGTCGTGATTGGCTCCGGTGCGACCGCCGTGACGCTGGTACCG
GCCCTGGCAGATTCCGGCGCTAAACACGTTACCATGCTGCAGCGTTCTCCGACTTATATCGTCTCTCAGCCAG
ACCGTGACGGTATCGCGGAAAAACTGAACCGTTGGCTGCCGGAAACCATGGCCTACACGGCAGTGCGTTG
GAAAAACGTCCTGCGTCAGGCGGCAGTGTATTCTGCTTGCCAGAAATGGCCGCGTCGCATGCGCAAAATGT
TCCTGTCCCTGATTCAGCGTCAACTGCCGGAAGGTTATGACGTACGCAAACATTTCGGTCCTCACTACAACCC
GTGGGATCAGCGTCTGTGTCTGGTACCGAACGGCGACCTGTTCCGCGCTATCCGTCATGGTAAAGTTGAAGT
AGTTACCGACACCATCGAACGCTTCACTGCTACCGGTATCCGCCTGAACTCTGGCCGCGAACTGCCGGCTGA
TATCATCATCACCGCTACCGGTCTGAACCTGCAGCTGTTTGGTGGTGCGACCGCCACCATCGACGGCCAGCA
GGTGGACATCACTACCACCATGGCATACAAAGGTATGATGCTGTCCGGCATCCCGAACATGGCTTATACTGTT
GGTTACACCAACGCGTCTTGGACTCTGAAAGCTGATCTGGTTTCCGAATTCGTGTGTCGTCTGCTGAACTATA
TGGACGATAACGGCTTCGATACCGTGGTGGTTGAACGCCCAGGTTCTGATGTTGAAGAGCGTCCGTTCATG
GAATTCACTCCAGGCTACGTACTGCGCTCTCTGGACGAACTGCCAAAACAGGGTAGCCGTACCCCGTGGCG
CCTGAACCAGAACTACCTGCGTGACATCCGCCTGATCCGTCGCGGCAAAATTGACGACGAAGGCCTGCGCT
TCGCAAAACGCCCGGCTCCGGTTGGCGTT

Figure S2. The optimized sequence of gene *EthA*.

Figure S3.

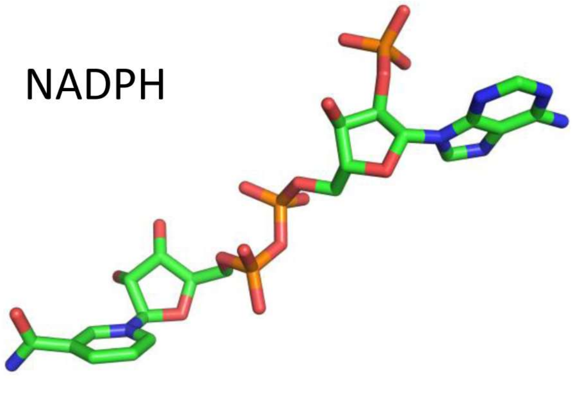

Figure S3. The chemical structure of NADPH (Nicotinamide adenine dinucleotide phosphate).

Figure S4.

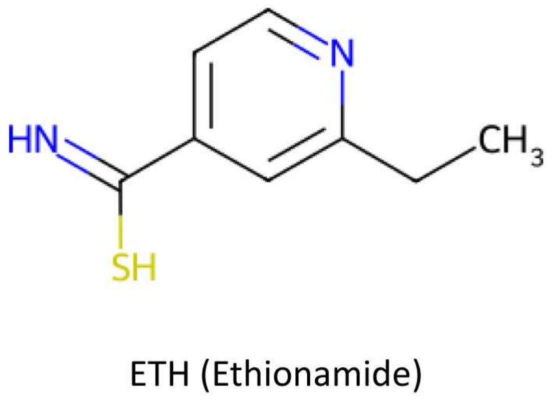

Figure S4. The chemical structure of ETH (Ethionamide).

Figure S5.

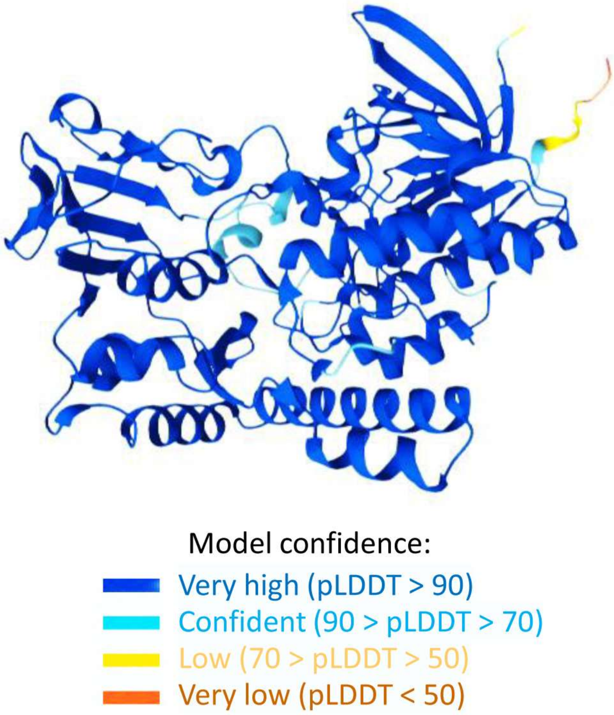

Figure S5. The confidence of the AlphaFold2-predicted EthA structure was evaluated using the per-residue Local Distance Difference Test (pLDDT), a 0-100 scale wherein higher scores denote greater predicted local accuracy.

Figure S6.

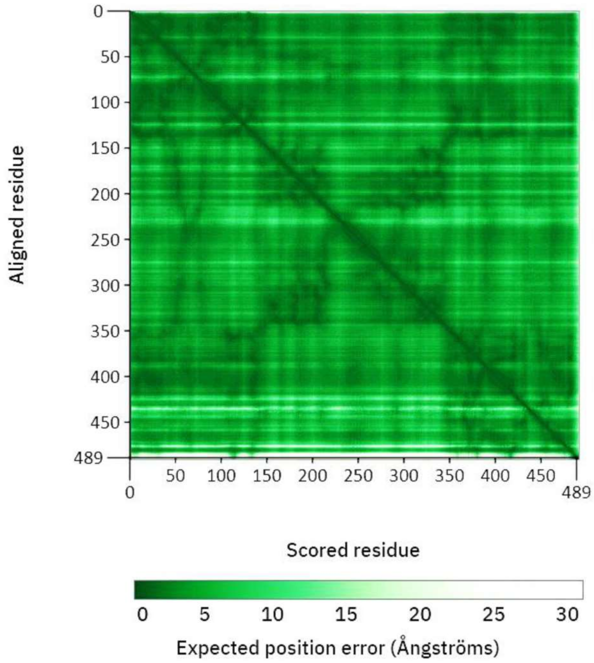

Figure S6. Predicted aligned error (PAE). PAE is a confidence metric generated by the AlphaFold system, representing the expected positional error between pairs of residues in the predicted structure. In the PAE plot, dark green denotes low error and high confidence, whereas orange or red indicates high error and unreliable relative positioning. The diagonal regions generally exhibit low error, reflecting stable predictions within individual domains. For off-diagonal regions, low error implies that the relative orientation and position between domains are accurately resolved; high error, conversely, suggests that AlphaFold is uncertain about the interdomain arrangement, and such results should not be used to infer domain-domain interactions.

Figure S7.

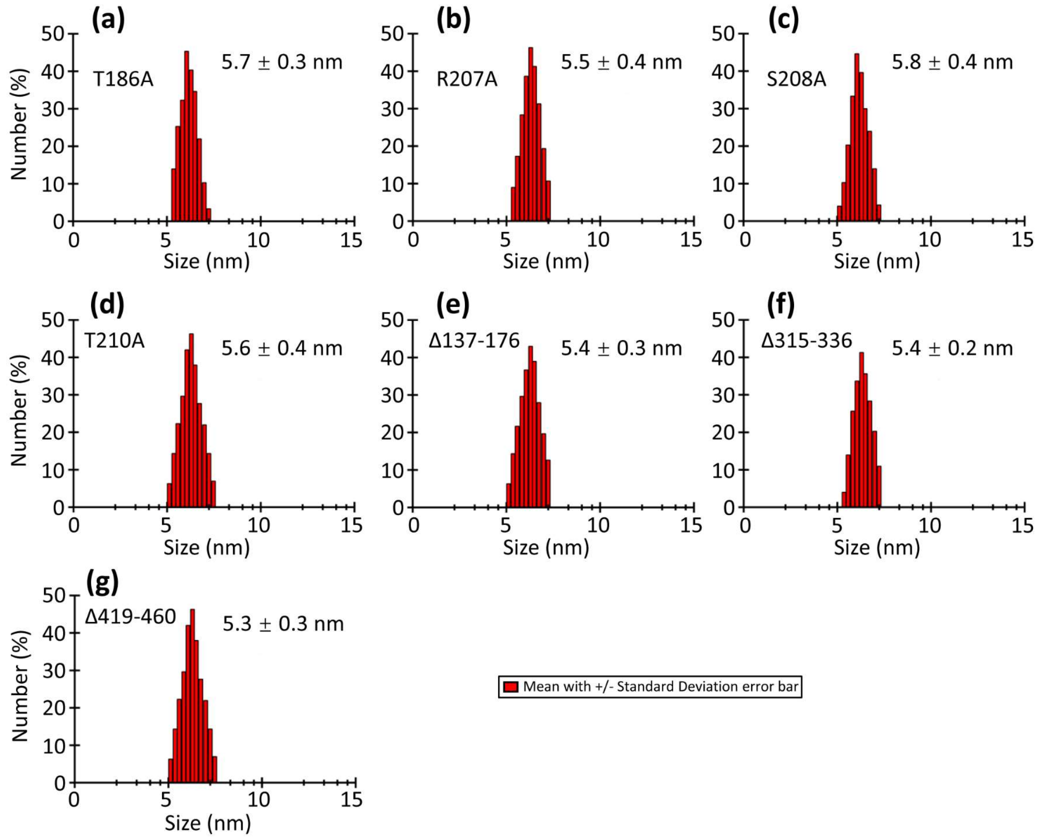

Figure S7. Dynamic light scattering (DLS) analysis of different EthA mutants. The hydrodynamic diameters of mutants R186A, R207A, S208A, T210A, Δ137-176, Δ315-336, and Δ419-460 were determined to be 5.7 ± 0.3, 5.5 ± 0.4, 5.8 ± 0.4, 5.6 ± 0.4, 5.4 ± 0.3, 5.4 ± 0.2, and 5.3 ± 0.3 nm, respectively, confirming that all variants remained in a monomeric state.

Table S1.

Table S1. Primers used for generating site-directed mutants of *EthA*

Primers	Primer sequence 5′-3′
T186A (F)	CGGTGCG**gca**GCCGTGACGC
T186A (R)	GTCACGGC**tgc**CGCACCGGAGCC
R207A (F)	CATGCTGCAG**gca**TCTCCGACTTATATCGTCTCTCAGCCAG
R207A (R)	GTCGGAGA**tgc**CTGCAGCATGGTAACGTGTTTAGCGC
S208A (F)	CTGCAGCGT**gca**CCGACTTATATCGTCTCTCAGCCAGACCG
S208A (R)	ATAAGTCGG**tgc**ACGCTGCAGCATGGTAACGTGTTTAGCGC
T210A (F)	GTTCTCCG**gca**TATATCGTCTCTCAGCCAGACCGTGACGGTATCGC
T210A (R)	GACGATATA**tgc**CGGAGAACGCTGCAGCATGGTAACGTGTTTAGCGC

Note: Mutagenic regions in the sequence are underlined and displayed in bold.

Table S2.

Table S2. Primers used for RT-qPCR

Primers	Primer sequence (5’-3’)
gyrA (F)	atgacagacacgacgttgccgcc
gyrA (R)	ttaattgcccgtctggtctgcgcc
EthA (F)	atgaccgagcacctcgacgttgt
EthA (R)	aacccccaccggggcag

Table S3.

Table S3. Dynamic light scattering parameters of purified EthA

Parameter	Value
Hydrodynamic diameter (D_h, nm)	5.6 ± 0.3
Polydispersity index (PdI)	0.08 ± 0.02
Diffusion coefficient (D_t, x 10^7 cm^2/s)	1.25 ± 0.06
Protein concentration (mg/mL)	2.6
Buffer composition	50 mM Tris-HCl (pH 7.5), 150 mM NaCl, 10% glycerol
Temperature (^oC)	25
